# Supplementary material for: Plasma Antithrombin III Levels Can Be a Prognostic Factor in Liver Cirrhosis Patients with Portal Vein Thrombosis
Source: Int J Mol Sci. 2023 Apr 23;24(9):7732. doi: 10.3390/ijms24097732 (PMC10178007; doi:10.3390/ijms24097732)
Supplement: Supplementary file 1 [file ijms-24-07732-s001.zip › ijms-2214400-supplementary.pdf]

Supporting Information Table S1. Comparison of patient characteristics between two groups with AT-III <54% and AT-III ≥54%, excluding HCC-related death.

	AT-III <54% (n=25)	AT-III ≥54% (n=36)
Age, years	66 (61–73)	68 (58–75)
Sex, male, n (%)	18 (72.0)	24 (66.7)
Etiology, HCV/HBV/Others, n (%)	12 (48.0)/0 (0.0)/13 (52.0)	18 (50.0)/7 (19.4)/11 (30.6)
HCC present, n (%)	10 (40)	8 (22.2)
Child–Pugh score	9 (7–11)	7 (6–8)
ALBI score	-1.41 (-1.69– -1.15)	-1.90 (-2.20– -1.61)

FIB-4 index	8.51 (5.5–12.2)	4.35 (2.22–9.48)
Alb (mg/dL)	2.8 (2.6–3.1)	3.2 (3.0–3.5)
T-Bil (mg/dL)	1.9 (1.5–2.7)	1.0 (0.7–1.5)
PT activity (%)	56 (47–65)	75 (63–86)

Data are presented as medians (interquartile ranges).

AT, antithrombin; HCC, hepatocellular carcinoma; HCV, hepatitis C virus; HBV, hepatitis B virus; ALBI, Albumin-Bilirubin; FIB-4, fibrosis-4; Alb, albumin; T-Bil, total bilirubin; PT, prothrombin time
